# Building a livestock genetic and genomic information knowledgebase through integrative developments of Animal QTLdb and CorrDB

**DOI:** 10.1093/nar/gky1084

**Published:** 2018-11-08

**Authors:** Zhi-Liang Hu, Carissa A Park, James M Reecy

**Affiliations:** Department of Animal Science, Iowa State University, 2255 Kildee Hall, Ames, IA 50011, USA

## Abstract

Successful development of biological databases requires accommodation of the burgeoning amounts of data from high-throughput genomics pipelines. As the volume of curated data in Animal QTLdb (https://www.animalgenome.org/QTLdb) increases exponentially, the resulting challenges must be met with rapid infrastructure development to effectively accommodate abundant data curation and make metadata analysis more powerful. The development of Animal QTLdb and CorrDB for the past 15 years has provided valuable tools for researchers to utilize a wealth of phenotype/genotype data to study the genetic architecture of livestock traits. We have focused our efforts on data curation, improved data quality maintenance, new tool developments, and database co-developments, in order to provide convenient platforms for users to query and analyze data. The database currently has 158 499 QTL/associations, 10 482 correlations and 1977 heritability data as a result of an average 32% data increase per year. In addition, we have made >14 functional improvements or new tool implementations since our last report. Our ultimate goals of database development are to provide infrastructure for data collection, curation, and annotation, and more importantly, to support innovated data structure for new types of data mining, data reanalysis, and networked genetic analysis that lead to the generation of new knowledge.

## INTRODUCTION

High-throughput genomics continues to rapidly generate a wealth of genome information for livestock animal species. Whole-genome assemblies for cattle ((1,2), chicken (3), pig (4), sheep (5), horse (6), catfish (7), rainbow trout (8) and other agricultural species have become available within the last 15 years. Efforts are ongoing to improve the quality of these assemblies and to functionally annotate gene information to them (9). Upon completion of functional annotation, the new genomes will provide powerful tools to study the genetic mechanisms that control traits of interest in livestock animals. Combined genetic and phenotypic correlation information from studies carried out in the past 70+ years, and quantitative trait loci (QTL) mapping results from studies over the past 25+ years, provide a huge amount of data that can be annotated to the genomes (10) and can help researchers elucidate the genetics underlying phenotypic variation. Our continued efforts in development of the Animal QTL Database (QTLdb) and Animal Trait Correlation Database (CorrDB) tools facilitate this process. It is worth noting that an average of over half a million annual web visits are made to Animal QTLdb (2010–2018), and there are >1680 references (including over 900 literature citations) to Animal QTLdb according to Google Scholar (https://www.animalgenome.org/QTLdb/publications), as of October 2018.

In genetic studies, QTL/associations are chromosomal regions that have been linked to complex traits by association analysis between polymorphic genetic markers and observed/measured phenotypic traits. Phenotypic and genetic correlations describe co-variations between traits with regard to livestock animal performance records and their genetic values. Originally, the Animal QTLdb and CorrDB were developed to house all relevant published data, with two primary functions: as a centralized repository for easy data retrieval, and as a platform for the comparison of data collected across different experimental, geographic, and methodological conditions (11–14). Throughout over 15 (QTLdb) and 7 (CorrDB) years, development of Animal QTLdb and CorrDB has evolved to take advantage of the demonstrated power and utility of resynthesis of metadata in terms of updated genetic analysis (13–16). This has fostered new opportunities and challenges in our developmental work as we strive to meet user demands and deliver QTL/association/correlation information in an easy to understand manner. The functions built into the databases allow queries of QTL/associations/correlations for genomic (genome locations, genes, related genome features and variations) and other types of associated data (e.g. pertinent studies, etc.), to provide networked views of the relevant genotypic and phenotypic information.

This report summarizes our most recent progress in development of Animal QTLdb and CorrDB, with a focus on synergistically reusing developed database components, combining functionalities, co-developing modules to integrate resources, and most importantly, providing genetic analysis tools that allow users to examine QTL/association-related data in a networked manner (Supplementary Table S1).

## NEW DEVELOPMENTS

### Accelerated data curation, improvement of data entry standards and well-managed database growth

**Figure 1.**
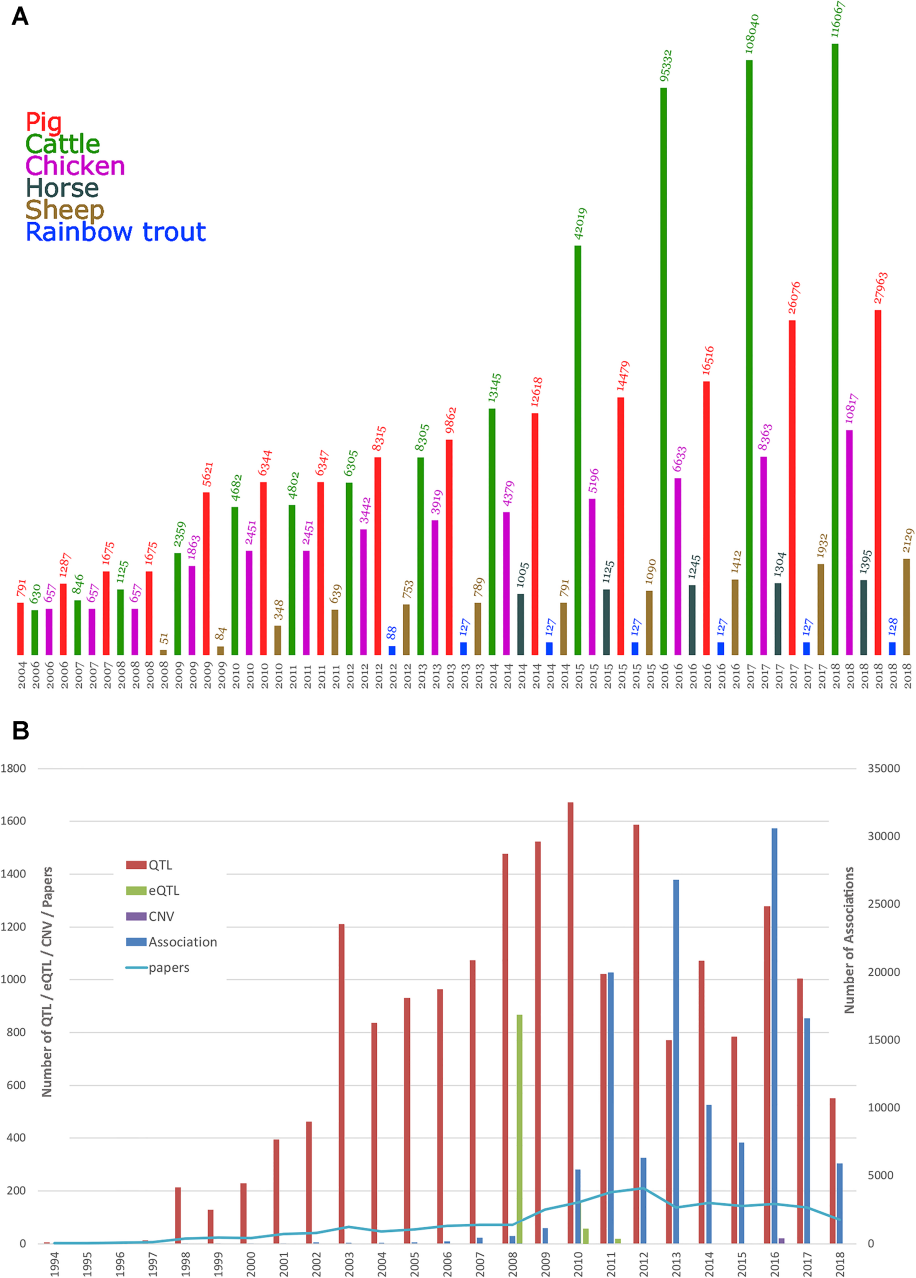
Growth of curated data in the Animal QTLdb by year and species, based on the total from three data releases per year on the Animal QTLdb website. Note that all data are log transformed so that the bar graphs can fit into a reasonable window size. (**A**) Animal QTLdb data growth by species. (**B**) Animal QTLdb data growth by data publication year and data type (note the different scale for the association data plot on the right axis). Please note that this is a reflection of data growth in the database, not a measure of all data in the public domain. There are data from recent years still in the curation pipeline that are not counted here.

The amount of curated data in the Animal QTLdb has undergone exponential growth over the past 15 years (Figure 1). To date, there have been 158 499 QTL/associations curated from 2040 journal articles that represent 1992 different traits in six livestock species (Figure 1A). Of all the included data types, the SNP association data have undergone the largest increase (Figure 1B).

Part of the curation process involves linking reported trait names with the best matches to corresponding standardized trait ontology terms, ideally while maintaining the ability to search for the names originally used in the literature. In addition, breeds/strains used in mapping, statistical evidence for linkage/association, as well as flanking markers for mapping in the genome, are added to link the trait to the genetic and/or sequence maps. This representation of the data in the database facilitates information transfer between different aspects of genetic analysis.

Data curation for the QTLdb and CorrDB is complicated not only by the ever-increasing volume of data that must be accurately processed, but also by the responsibility to maintain the entered data for the entirety of their lifetime in the databases. We have previously reported on the development of tools to ensure high-quality curation workflows (14); we have also generated a list of minimum required information for QTL/association data entry (https://www.animalgenome.org/QTLdb/doc/minfo). This helps to bring together the curators and database developers in order to minimize the gaps between collaborative curation efforts. Besides the existing curation protocols and data flow framework, we also provide a step-by-step guide to help data authors submit their data via our web tools. This gives them the opportunity to take ownership and manage their own data, and they can also upload their data in batch form. That process has been integrated to merge with our internal data curation flow (https://www.animalgenome.org/QTLdb/doc/batchdata).

### Implementation of tools for whole-genome analysis of QTL/association enrichment

Gene Ontology (17,18) enrichment analysis of large gene expression experiments has been recognized as an effective method for investigators to increase the likelihood of identifying biological processes most pertinent to their studies (19). The method has been well described (20). At an abstract level, a gene represents a region of the genome, as does a QTL. Similarly, gene ontology terms have been associated with genes as phenotypes are with QTL. Therefore, we wondered if it is possible to evaluate for over-enrichment of a phenotype/trait in regions of the genome. As an initial trial, we evaluated methods for a simple procedure to assess the enrichment of QTL/association data curated into the QTLdb with Chi-square analysis of a two-way contingency table (traits by chromosomes). Our current tool was designed to allow evaluation of all reported QTL/associations for selected traits throughout a genome, to determine if the trait or traits are over-represented in one or more regions of the genome. The setup of the analysis is based on an underlying assumption that the selected traits are related. For example, the traits may belong to the same trait type, or are from a given trait ontology branch.

**Figure 2.**
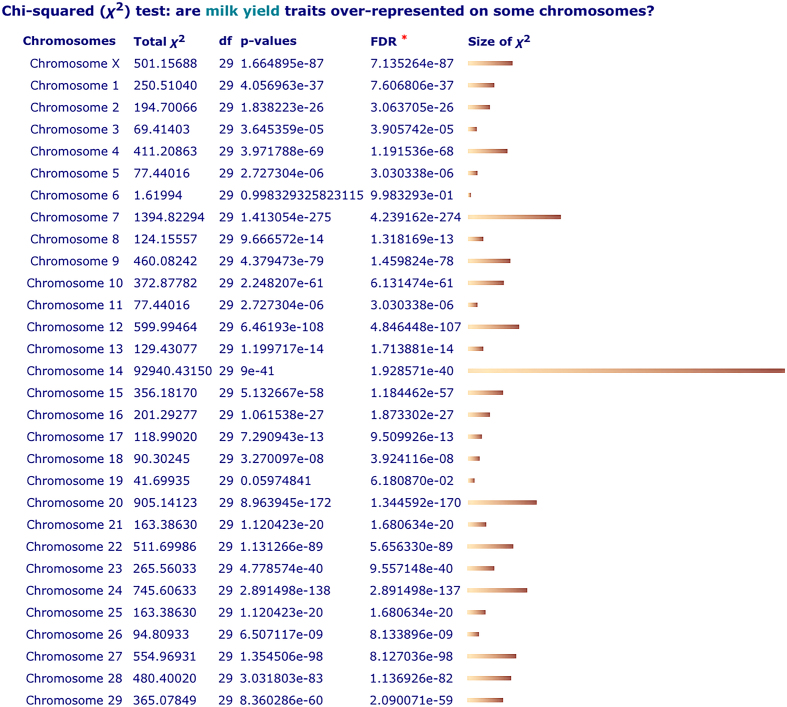
Example output from the QTLdb data enrichment analysis tool. The analysis was performed on 3827 ‘milk yield’ QTL/associations found in cattle (these milk traits represent a collection of seven related traits measured/estimated with different methods, each describing certain aspects of the ‘milk yield’). A Chi-squared analysis was performed on a 7 × 30 contingency table. The results show p-values for each chromosome, along with false discovery rate (FDR) values estimated using the Benjamini–Hochberg procedure. The transformed values of Chi-squares are plotted using horizontal bars to indicate locations where larger numbers of QTL/associations are found.

Figure 2 shows the output from our initial implementation, in which 3827 ‘milk yield’ QTL/associations, representing seven related traits and found on 30 cattle chromosomes, are ‘enriched’ in certain chromosomal locations. We used Chi-square analysis of the frequencies of reported QTL/associations classified by traits and chromosome locations. The contingency p-values (p) are estimated to indicate the degree of over-representation (enrichment) of QTL/associations. The false discovery rate (FDR) is estimated using the Benjamini–Hochberg procedure (21). The sizes of Chi-squares in each contingency category are graphically plotted with bars of varying lengths to indicate the locations where larger numbers of QTL/associations are found. Efforts are still under way to implement additional functionality allowing analysis of user-defined chromosomal sub-regions and selection of trait sets.

Currently, trait correlation data are appended to the enrichment report when available, to provide additional supporting information to help users to evaluate the results (not shown here but available online). This demonstrates that the potential exists for more complex enrichment analysis involving more networked factors.

### Integrated development of VT/LPT/CMO ontologies and their mapping to traits maintained within QTLdb/CorrDB

**Figure 3.**
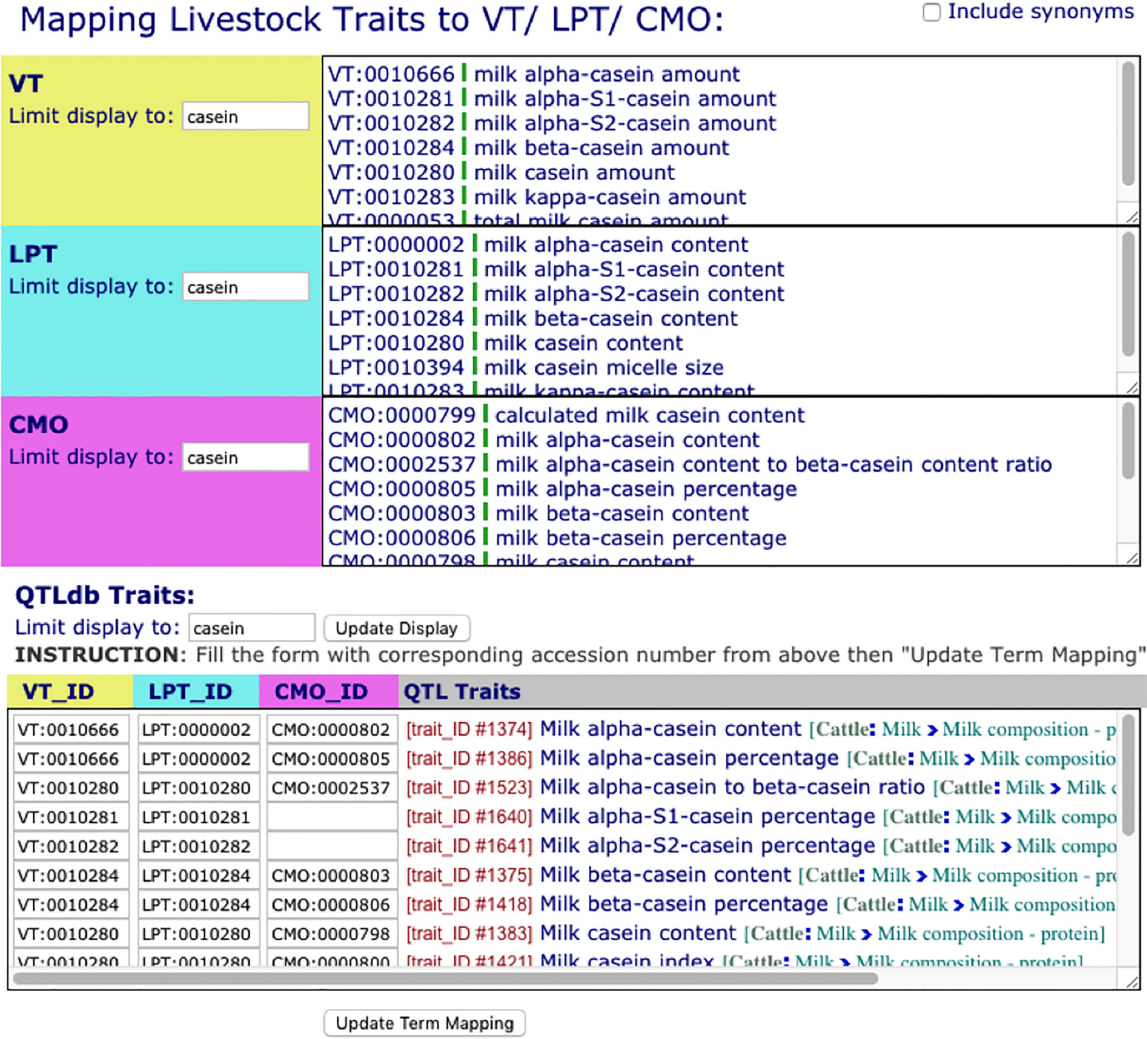
A screenshot of the trait mapping tool used in the Animal QTLdb and CorrDB. The tool accommodates comparative views of the three ontologies against the livestock trait set, so that the best match can be chosen while providing feedback for ontology developments.

Among livestock producers and genetics researchers, the naming of traits is highly variable. A strategy for unambiguous management of these data during database development is the use of biological ontologies. We have previously described (14) how the Vertebrate Trait Ontology (VT; https://www.animalgenome.org/bioinfo/projects/vt/; (22)), Livestock Product Trait Ontology (LPT; https://www.animalgenome.org/bioinfo/projects/lpt/), and Clinical Measurement Ontology (CMO; https://www.animalgenome.org/bioinfo/projects/cmo/; (23)) are used to annotate traits within Animal QTLdb. Now these three ontologies are also being used to manage traits for the Animal CorrDB. In order to accomplish this, we developed a trait mapping tool (Figure 3) to map traits for CorrDB, with the goal of a unified system of trait management in both QTLdb and CorrDB. The mapping tool brings three ontology lists and one target (QTLdb or CorrDB) trait list into the same viewing frame, with each list searchable and scrollable, so that targeted comparisons can be made and the best matches identified within one window. This view also provides a way for ontology developers to make comparisons among similar terms from different ontologies, thus creating feedback information for the fine-tuning of ontology development.

**Figure 4.**
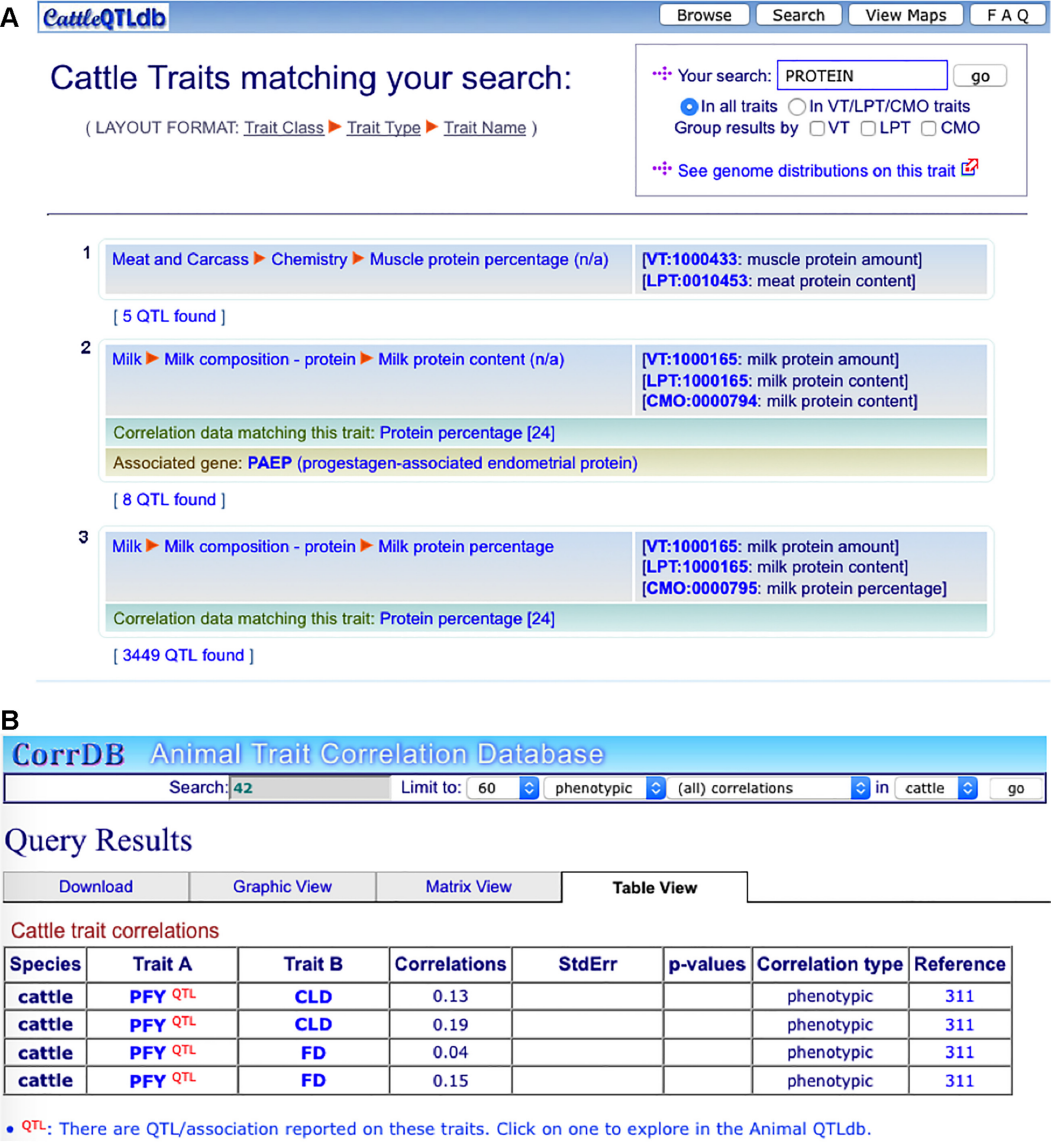
Data links between the Animal QTLdb and CorrDB are achieved based on their mutual trait mapping to VT/LPT/CMO ontologies. (**A**) A QTL/association data view showing links to CorrDB where they exist (highlighted in light green). (**B**) A CorrDB view of correlations showing traits with existing QTL/association data.

The ontological integration of traits from the QTLdb and CorrDB enables dynamic data links between the databases. Figure 4 includes two screenshots, showing that in QTLdb, when correlation data is available, they are provided on a QTL/association data sheet listed by trait (Figure 4A); and in CorrDB, available corresponding QTL/association data are provided on a correlation data table view (Figure 4B).

### Managing new types of traits using ‘modifiers’ as additional trait attributes

**Figure 5.**
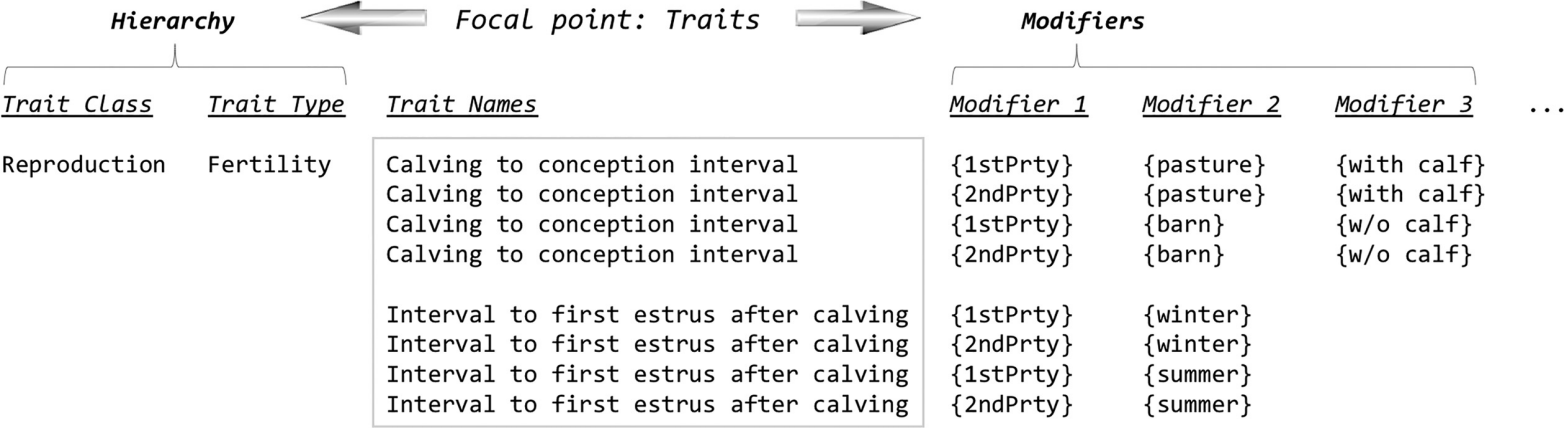
A diagram showing how the ‘modifiers’ are used to annotate traits in the environment of ontology management of hierarchies. Use of the modifiers effectively allows multidimensional attributes to be appended to a trait.

**Figure 6.**
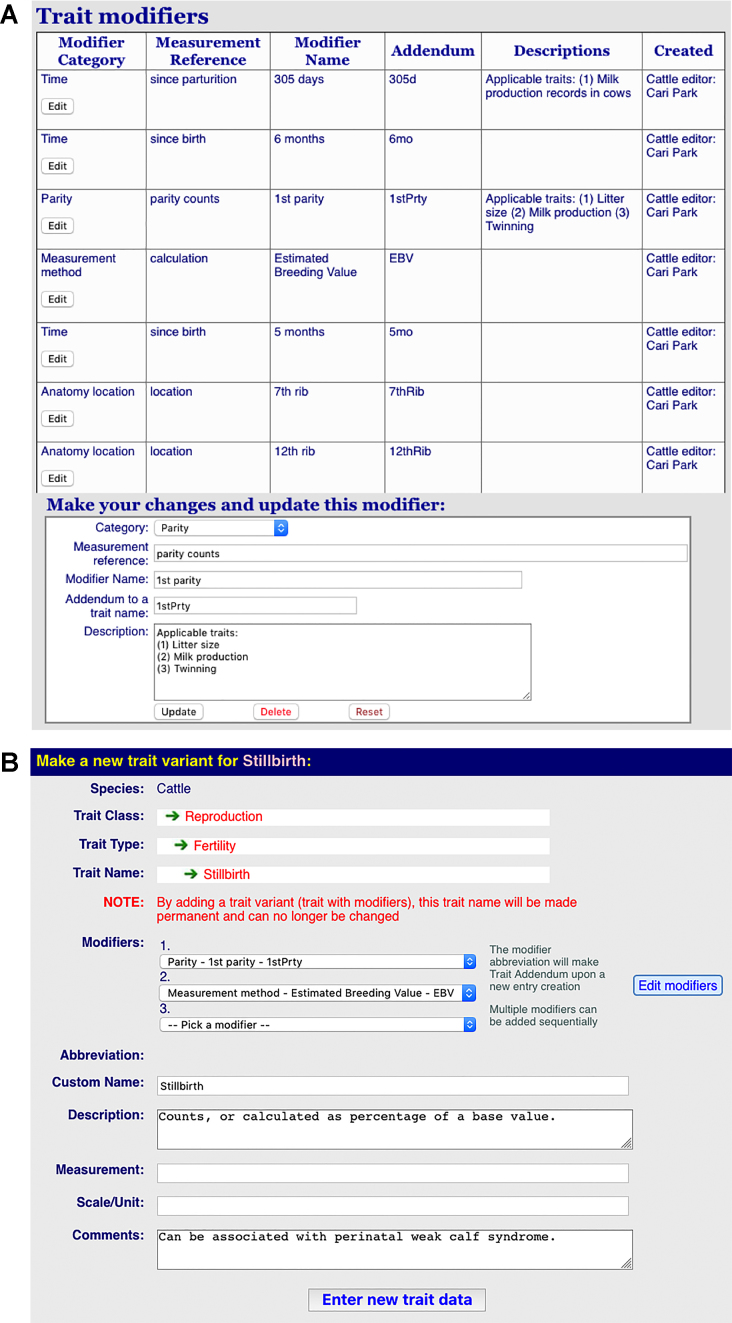
Screenshots of the trait editor tools showing how trait modifiers are managed with controlled vocabulary and context (**A**), and the formulation of a trait variant by adding modifiers to more clearly define how a trait may be evaluated in a given context (**B**). (A) A QTLdb editor window showing how ‘modifier’ attributes are managed. (B) A QTLdb editor window showing how a trait variant with modifiers can be created.

A new challenge we have encountered is that in many correlation reports, the number of trait name variations is so high that it causes difficulty for typical curation workflow and effective trait data management. The naming variations mostly come from how or when traits are measured. For instance, pig litter size can be measured at the sow's first parity (‘first parity litter size’), second parity (‘second parity litter size’) or later; and backfat thickness may be measured by ultrasound or ruler, as well as at different locations along the back/ribs, etc. In order to enable meaningful comparisons between the data behind these traits, while bringing a potentially overwhelming number of similar traits to a reasonable level for routine curation, we introduced the use of ‘modifiers’ to trait names as an attribute, while keeping the list of ‘base trait’ names at a manageable size. These modified traits may be treated as separate entities for the sake of comparison, but in ontology terms, they would all be annotated to the same trait. Figure 5 shows a conceptual diagram that describes how ‘modifiers’ are used to attach attributes to traits in a hierarchical system. In a newly implemented process, we allow ‘modifiers’ to alter traits based on measurement methods, time, anatomical locations, etc., while still retaining the original definition as a base trait for ontology data management. Currently we call this type of modified traits ‘trait variants.’ To facilitate the process, we have been developing a list of ‘modifiers’ with their own controlled vocabulary (Figure 6A). These modifiers can be appended to a trait, making the expanded term distinguishable from other variants, yet retaining the base trait in its original form (Figure 6B). Multiple modifiers may exist; each modifier captures a particular aspect of a trait in which the underlying concept can have different interpretations (24). With multiple modifiers, a trait concept can be compounded. Work is underway to characterize these new trait variants.

### Gene-centric and trait-centric views of QTL/association data

**Figure 7.**
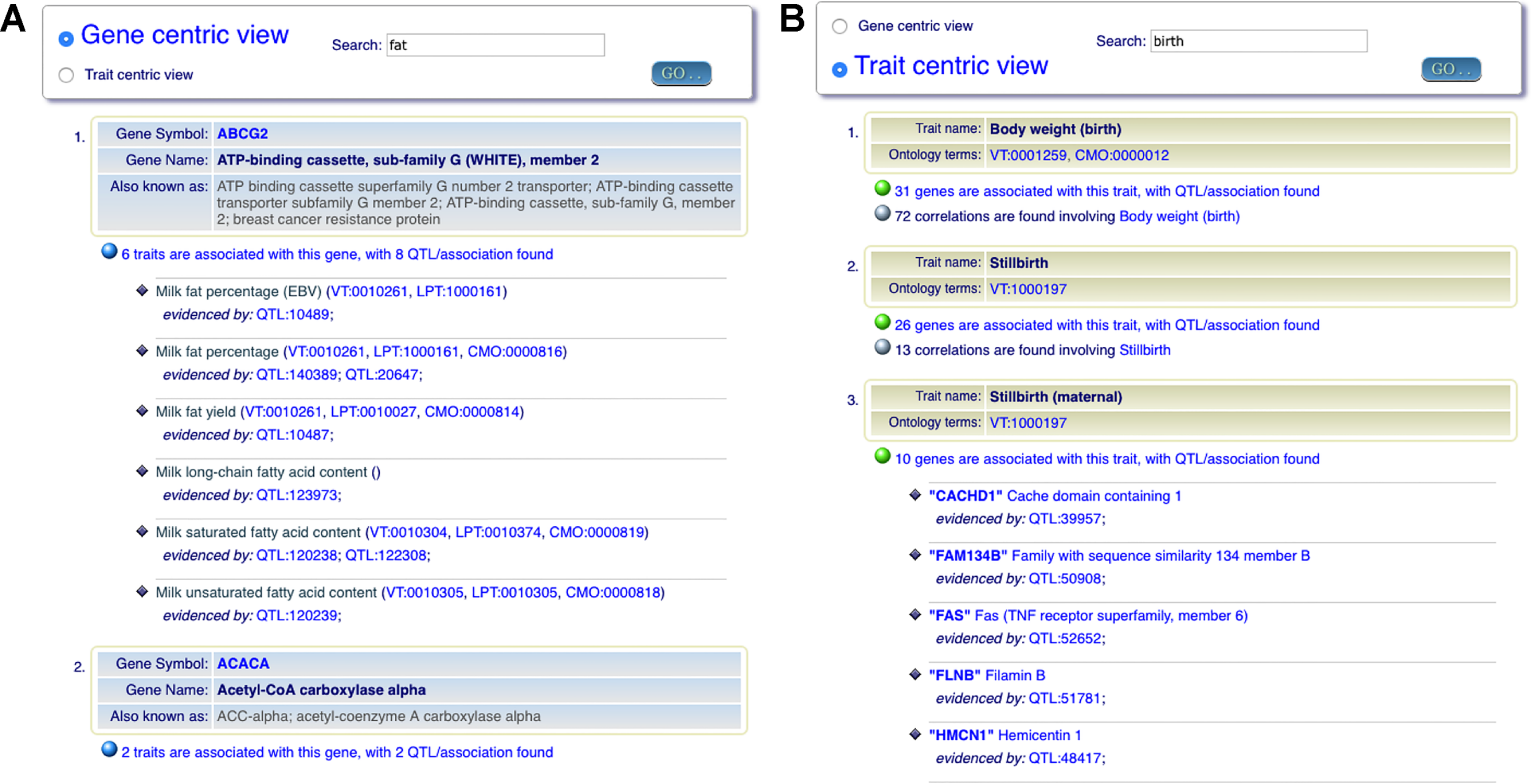
Gene-centric (**A**) and trait-centric (**B**) views of animal QTL/association data. Note that the long QTL/association list is hidden upon first loading of the page. This allows users a quick view of the gene or trait list before expanding the details on a particular item for closer examination or data download. (A) Gene-centric view of lists of QTL/association data. (B) Trait-centric view of lists of QTL/association data.

With the exponential data growth in QTLdb, it is necessary to maintain the ability to quickly extract relevant genotype-phenotype information for human-consumable analysis. To this end, we have developed new tools to digest the data into gene-centric and trait-centric views by organizing information linked to genes (or traits), making it easier for users to follow the information flow. Two screenshots are shown, in Figure 7A and B, that demonstrate how gene-centric and trait-centric views of the QTL/association data are displayed. For example, upon user query, a list is generated with summaries of gene name, symbol, and any other known names. Dynamic links to NCBI GeneDB are embedded to provide more detailed information on each gene, and the display also indicates the number of QTL/association data that are associated with the gene. Options are also available for users to open up the QTL/association data list for exploration or for download. Likewise, the traits on a trait-centric view have dynamic links to VT/LPT/CMO ontology terms when such mapping exists. We also made it possible for users to browse for information of interest and download the data from a web snapshot.

### Additional or supplementary data

With high-throughput data analysis, many publications come with additional data, either as additional information to support the findings, or as supplementary data to show near-significant results. Such data may not meet a journal's publication requirements or be essential for a publication, but they are collectively useful for future meta-analysis. We have extended the database structure to allow this data to be directly uploaded as they are, with an information link appended to the released data. Normally these data include original genotypes, phenotypes, near-significant or sub-optimal association/QTL data from the same experiment, supplementary to the curated (significant) data. Currently this type of data cannot be directly accessed by the public but is available upon request. We include these data in an attempt to host more complete data collections to improve future metadata analysis.

### Improved procedures and tools for data release

Based on our past practice and experiences, we have developed an extensive process for data quality control and review before curated data may be released to the public (Supplementary Figure S1; Supplementary Table S2). Although we have tried to automate these steps as much as possible, currently about 60% of the operations are still manual or semi-manual, partly because human attention is required for situations where scripts must be used with care in order to properly and safely handle the exceptions frequently caused by data variations. Often, additional communication is needed between the administrator/editor and curator to obtain additional information or to confirm data status. In addition, post-curation and post-release data debugging is becoming part of our routine as curation activities increase and unusual data situations arise.

Several new curation procedures/protocols have been developed since our last report (14). Data may now be ‘*re-tracked*’ for valid reasons; placed ‘*on hold*’ when verification is needed due to contradicting or confusing information; ‘*suspended*’ when problems are found; and ‘*obsoleted*’ in cases where erroneous, duplicate, or problematic data is identified (data is not physically deleted from the database). Under certain circumstances we may also conditionally release data, for example, to accommodate authors’ requests to pre-release a set of data to meet publication requirements. In addition, cron jobs have been implemented to automatically prompt curators/authors of an ‘interim’ data set for new updates. This helps to avoid leaving unfinished data behind.

### Other developments

There are a few relatively minor and independent tool developments worth mentioning:

- We developed a ‘permanent record locator’ as a unique identifier specific to a set of released data, often of a paper, report or author(s). These unique identifiers are permanent IDs linked to a complete list of QTL/association/correlation data points from the same source. The record locators can be embedded in URLs to create dynamic web addresses that may be used by data authors to present to journal editors/reviewers as proof of database entry, for external reviews of data quality, and for use on their websites to directly link to the data.
- Improved curation tools allow SNP information with ‘rs’ number to be brought directly from external databases instead of being added manually.
- An improved batch data entry tool allows tabulated data prepared in an Excel sheet to be loaded ‘as is’ with column selectors without the need for tedious manual formatting. This has proven to be an efficient way to incorporate data from additional data sheets of a publication, sparing curators from laborious manual efforts.
- Improved curation tools now allow ‘ss’ SNP IDs to be entered. This is important because there is lag time between the submission of a set of new SNPs to the European Variation Archive (EVA; for non-human data previously housed by dbSNP) and the assignment of the official ‘rs’ ID numbers when all SNPs have been validated and accepted.

## DISCUSSIONS

The QTLdb was originally developed to house all published results of QTL studies, allowing users to make comparisons across experiments (Hu *et al.*, 2007). New and improved tools have made the database a valuable resource for meta-analysis and data reuse. In the era of big data and high-throughput technologies, the extended utility of the QTLdb has become more evident with the growth of not only the amount of data but also data types such as SNP association, copy number variations, expression QTL (eQTL), haplotypes, etc. With the co-development of CorrDB, trait correlation and heritability data add new dimensions to elucidate the genetic architecture. Essentially, the development of QTLdb and CorrDB opens the door to genetic network analysis with multiple factors. The newly added gene/trait enrichment analysis is one of the tools that helps us achieve our goal to develop QTLdb/CorrDB into a centralized knowledgebase.

The functionalities we develop for both QTLdb and CorrDB provide platforms that are not only useful for stable data storage, management, and quick retrieval, but also for data processing and analysis, even for large amounts of data. Improved computational capabilities have made collection, processing, presentation, and analysis of such data possible in a reasonable time frame. Our efforts to develop the Animal QTLdb/CorrDB for structured data collection provide valuable groundwork to fuel future metadata analysis. With the amount of genotype/phenotype association data being published at an accelerating rate, this work will increase possibilities for us to harness big data to better comprehend the relationships between livestock genotypes and phenotypes.

Compared to our previous reports on Animal QTLdb development, this report focuses more on back-end data dissections, curation improvements, process fine-tuning, and database management with new tools developed to serve these purposes. One challenge we have been undertaking is the elimination of data handling bottlenecks. For example, some of our improvements have included finding better solutions for query strategies, algorithms, database and data structures, and hardware to query SNP data in a practical manner considering the hundreds of millions of rows of data handled as part of our daily curation routine.

Currently, the Animal QTLdb and CorrDB each appear to have their own external web interface and environmental settings. However, it is our goal and development practice to integrate them under one system with seamless data/function connections for improved data flow. With this in mind, we are co-developing tools, data routines, and database structures using shared developmental resources. Looking forward, an important goal of these efforts is to develop structured data collections to expand our ability to facilitate future meta-analysis and genetic network analysis. There are always gaps between the data that would optimally be curated and the data that is available for us in the public domain. This presents challenges for our developmental efforts as we strive to collect all possible data. Continued data accumulation adds new potential for future data analysis; however, structured data collection, and the ability for data to be ‘re-synthesized,’ adds power for improved meta-analysis in the future. It is our hope that we will gradually close these gaps and be able to help maximize the utility of genotype-to-phenotype data to ultimately address issues important to the livestock industry.

## DATA AVAILABILITY

The database contents and tools are all freely available online. QTLdb: https://www.animalgenome.org/QTLdb/; CorrDB: https://www.animalgenome.org/CorrDB/. In addition, the data is also available upon release at several data alliance partner websites, including NCBI: http://www.ncbi.nlm.nih.gov/gene; Ensembl: http://www.ensembl.org/; UCSC: https://genome.ucsc.edu/cgi-bin/hgGateway; Reuters Data Citation Index: http://wokinfo.com/products_tools/multidisciplinary/dci/.

## Supplementary Material

Supplementary Data
