# Supplementary material for: Building a livestock genetic and genomic information knowledgebase through integrative developments of Animal QTLdb and CorrDB
Source: Nucleic Acids Res. 2018 Nov 8;47(Database issue):D701–10. doi: 10.1093/nar/gky1084 (PMC6323967; doi:10.1093/nar/gky1084)
Supplement: Supplementary Data [file gky1084_supplemental_files.zip › Figure S1.docx]

| **Figure S1. A Curator/Editor/Administrator Workflow for Data Curation and Management within the**  **Animal QTLdb and CorrDB** |
| --- |
| 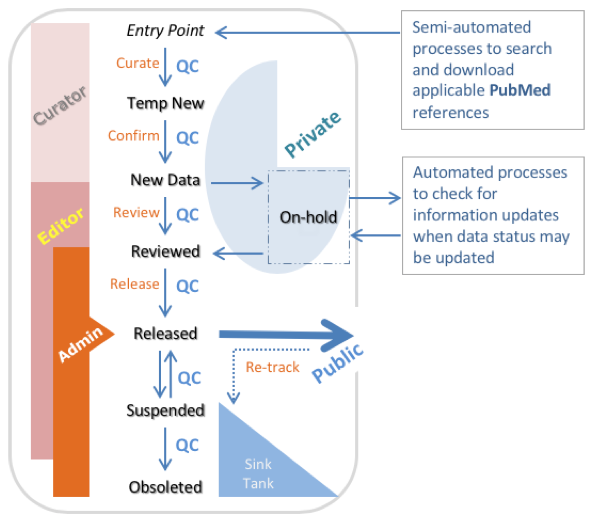 |
