# Supplementary material for: Building a livestock genetic and genomic information knowledgebase through integrative developments of Animal QTLdb and CorrDB
Source: Nucleic Acids Res. 2018 Nov 8;47(Database issue):D701–10. doi: 10.1093/nar/gky1084 (PMC6323967; doi:10.1093/nar/gky1084)

Table S1.
Animal QTLdb and CorrDB data release procedures and check items.

Steps	High-lights	Operatioins
**Step 0**:<br>Overview/routine monitor of new data in the curation pipeline	Spot checks of data with admin web tools<br>Monitor data flow/motion<br>Monitor for missing data (essential data such as statistics), wrong data range (such as map locations), etc.<br>Monitor "left behind" data by curators (unfinished entries)<br>Communicate with curators for problems identified	Overview/monitor of data flow with a set of administrator web tools<br><br>This is part of the routine of the DB admin prior to the database release stage.
**Step 1**:<br>Run check points	Re-populated 'breed' table with QTL/association information.<br>Update gene info from NCBI (where only Gene ID is curated)<br>Check for any missing statistics<br>Check any missing map info.<br>Check if SNPs are available where coordinates are manually entered.<br>Fix 1: Populated empty coordinates fields where SNP is available<br>Fix 2: Convert 'bp' to 'cM' where applicable<br>Fix 3: Fill 'peak'/'span' by their linkage marker locations<br>Fix 4: Convert 'cM' to 'bp' where applicable<br>Fix 5: Fill missing symbols/names in QTLdata table<br>Fix 6: Find and fix inverted bp locations	Each operation is aided with scripts specifically developed for each specific purpose. Operations require human verification of input/output/error report to ensure valid processes, identify new problems, exceptions. Modify scripts for fixes where apply.

	Fix 7: Look for 'rs' number of 'ss' SNPs<br>Fix 8: Find missing or conflict QTL Symbols<br>Fix 9: Run map liftover when applicable	
**Step 2**:<br>Verify new reference PDF files	Find all physical PDF files, “touch” db<br>Identify missing PDF files, “touch” db<br>Move PDF file in place from upload pool; Check for errors.	This is to make the backend links of curated data to their sources (PDF files where the data were published) for future data quality control checkups.
**Step 3**:<br>Do the "release"	Database: List data by curators, species, verification status<br>Web site: Publish release statistics<br>Release summary: Compose release data summary	Run scripts; Issue option to release; Log automatically kept<br>Semi-automated data updates on web<br>Add tools update descriptions
**Step 4**:<br>Post-release operations	Prepare data for download<br>for NCBI (pre-agreed data format)<br>for Routure (pre-agreed data format)<br>for UCSC (pre-agreed data format)<br>for Public users (with updated format)<br>GBrowse: Re-set up<br>JBrowse: Re-set up<br>Biomart: data re-import<br>Intermine: (internal developments)	Data refresh on portals of our data alliances and third party tools.

**Step 5**:<br>Post-release updates	Update “QTL Gene” IDs from NCBI	To complete the new QTL/association data entries with "Gene IDs" assigned by NCBI GeneDB.

Figure S1.
A curator/editor/administrator workflow for data curation and management within the Animal QTLdb and CorrDB

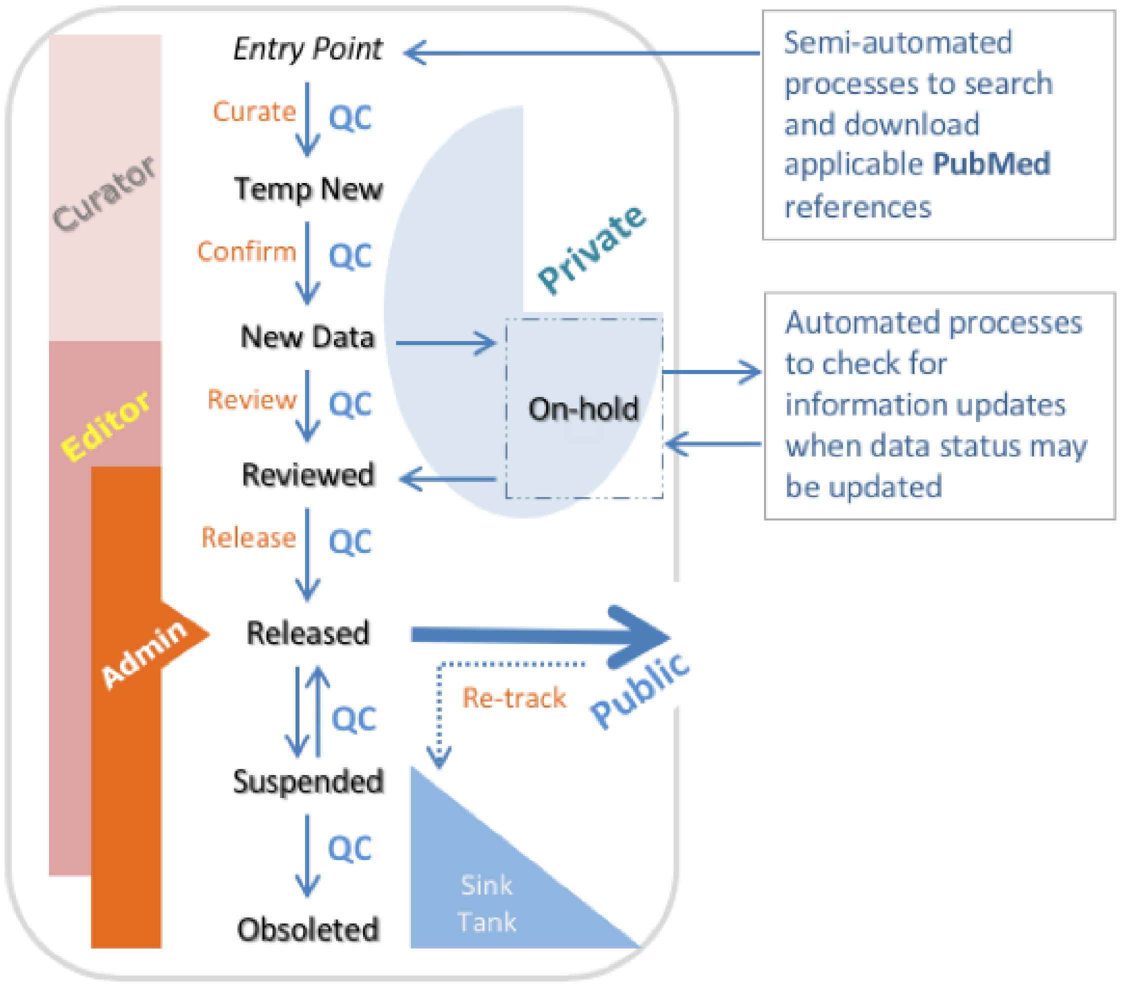

Supplement: Supplementary Data [file gky1084_supplemental_files.zip › Table S1 and Figure S1.pdf]
