# Supplementary material for: Building a livestock genetic and genomic information knowledgebase through integrative developments of Animal QTLdb and CorrDB
Source: Nucleic Acids Res. 2018 Nov 8;47(Database issue):D701–10. doi: 10.1093/nar/gky1084 (PMC6323967; doi:10.1093/nar/gky1084)
Supplement: Supplementary Data [file gky1084_supplemental_files.zip › Table S1.docx]

**Table S1.
New or Improved Functoinalities on Animal QTLdb and CorrDB since Our** [**2016 Report**](https://academic.oup.com/nar/article/44/D1/D827/2502740)

| Items | Functions | Details |
| --- | --- | --- |
| 1. | Supplementary data, not essential but attached to a publication, can be uploaded "as is". | These data include original genotypes, phenotypes, near-significant or other association/QTL data from the same experiment, supplementary to the curated data. We are still in the process of developing schemes where the use of these data by the public or third parties can best be managed. |
| 2. | New file format templates are developed pursuant to the "Minimum information required for Animal QTLdb data entry" in the form of Excel work sheets. | On the [minimum information](https://www.animalgenome.org/QTLdb/doc/minfo) page, click the icon on the right hand side to see an example, and scroll to the bottom of the page to download all Excel templates. More such templates are still being added to provide a convenient way for users to prepare their data for upload into the QTLdb |
| 3. | The curation tools are improved to allow automatic verification and streamlined curation of SNP information into the database. | The improvements involved data quality control processes in which multiple genome builds are maintained for cattle, chicken, and sheep. |
| 4. | Preliminary SNPs (with 'ss' numbers only) are allowed to be entered to anchor QTL/association mapping locations | The QTL/association mapping data represented by the 'ss' SNPs are not subject to normal data release procedure unless the manually entered map coordinates are also available. The 'ss' SNP information will eventually be replaced with their 'rs' SNP information once they are assigned by dbSNP. |
| 5. | A "permanent record locator" has been implemented in the AnimalQTLdb to provide unique and stable links to all curated QTL/association data for a publication | The new "permanent record locator" is a replacement of a "URL Link" function introduced two years ago and overcomes some of its shortcomings. It can be used by the authors to refer to their published data, to provide proof of evidence of data entry at the Animal QTLdb to journal editors/manuscript reviewers, and to serve other purposes. All QTLdb curators/editors have access to this tool for their curated data. |
| 6. | Periodic data checks on "on-hold", "conditionally released" (such as QTL/associations marked with 'ss' SNPs), and "suspended" data have been automated. | This helps to make sure no sub-optimal data goes unnoticed. |
| 7. | New set of curator tools for the Animal Correlation Database (CorrDB) | Includes new forms/routines for improved experiment information entry, new correlation data entry, and new measurement data entry. |
| 8. | New functions to link QTL data with trait correlation data through common trait terms where such data exist. | For example, within the CorrDB, under the Table View of correlation data, a hyper-linked red "QTL" label is added when QTL data exists on the trait; Like-wise, within the QTLdb on the trait search results, an hyper-linked tag is added on traits where correlation data are found |
| 9. | New "gene centric view" and "trait centric view" of QTL/association data where underlining gene information is available. | This tool helps users to relatively quickly to interrogate and understand genotype-to-phenotype information in a synopsis. A quick way to use the tool to view information is to try a search by genes or by traits. |
| 10. | New capacity to house information for and management of trait variants characterized by modifiers. | This is to manage new types of traits using “modifiers” as additional trait attributes. For example for the "same" traits by nature but slightly different in terms of attached information, such as time, location, and other conditions as "modifiers". |
| 11. | Customized structure modification to support two genome maps for cattle. | Although the initial implentation was for cattle, similar mechanism will work for other species. |
| 12. | Data flow was modified to allow data retract (withdraw). | Retract was implemented on two levels: Individual data level and publication (paper) level (for group withdraw). |
| 13. | QTL/associations data enrichment analysis tool. | A simple procedure to assess the enrichment of QTL/association data curated into the QTLdb with Chi-square analysis of a two-way contingency table (traits by chromosomes). Our current tool was designed to allow evaluation of all reported QTL/associations for selected traits throughout a genome, to determine if the trait or traits are over-represented in one or more regions of the genome. |
| 14. | Tools for ontology mapping between VT/LPT/CMO terms and QTLdb/CorrDB traits. | Targeted comparisons can be made and the best matches identified. This also provides a way for ontology developers to make comparisons among similar terms from different ontologies, thus creating feedback information for the fine-tuning of ontology development. |
