# Supplementary material for: Building a livestock genetic and genomic information knowledgebase through integrative developments of Animal QTLdb and CorrDB
Source: Nucleic Acids Res. 2018 Nov 8;47(Database issue):D701–10. doi: 10.1093/nar/gky1084 (PMC6323967; doi:10.1093/nar/gky1084)
Supplement: Supplementary Data [file gky1084_supplemental_files.zip › Table S2.docx]

**Table S2.
Animal QTLdb and CorrDB Data Release Procedures and Check Items.**

| Steps | High-lights | Operations |
| --- | --- | --- |
| **Step 0**:   Overview/routine monitor of new data in the curation pipeline | 1. Spot checks of data with admin web tools 2. Monitor data flow/motion 3. Monitor for missing data (essential data such as statistics), wrong data range (such as map locations), etc. 4. Monitor "left behind" data by curators (unfinished entries) 5. Communicate with curators for problems identified | Overview/monitor of data flow with a set of administrator web tools  This is part of the routine of the DB admin prior to the database release stage. |
| **Step 1**:   Run check points | 1. Re-populated 'breed' table with QTL/association information. 2. Update gene info from NCBI (where only Gene ID is curated) 3. Check for any missing statistics 4. Check any missing map info. 5. Check if SNPs are available where coordinates are manually entered. 6. Fix 1: Populated empty coordinates fields where SNP is available 7. Fix 2: Convert 'bp' to 'cM' where applicable 8. Fix 3: Fill 'peak'/'span' by their linkage marker locations 9. Fix 4: Convert 'cM' to 'bp' where applicable 10. Fix 5: Fill missing symbols/names in QTLdata table 11. Fix 6: Find and fix inverted bp locations 12. Fix 7: Look for 'rs' number of 'ss' SNPs 13. Fix 8: Find missing or conflict QTL Symbols 14. Fix 9: Run map liftover when applicable | Each operation is aided with scripts specifically developed for each specific purpose. Operations require human verification of input/output/error report to ensure valid processes, identify new problems, exceptions. Modify scripts for fixes where apply. |
| **Step 2**:   Verify new reference PDF files | 1. Find all physical PDF files, “touch” db 2. Identify missing PDF files, “touch” db 3. Move PDF file in place from upload pool; Check for errors. | This is to make the backend links of curated data to their sources (PDF files where the data were published) for future data quality control checkups. |
| **Step 3**:   Do the "release" | 1. Database: List data by curators, species, verification status 2. Web site: Publish release statistics 3. Release summary: Compose release data summary | 1. Run scripts; Issue option to release; Log automatically kept 2. Semi-automated data updates on web 3. Add tools update descriptions |
| **Step 4**:   Post-release operations | 1. Prepare data for download    - for NCBI (pre-agreed data format)    - for Routure (pre-agreed data format)    - for UCSC (pre-agreed data format)    - for Public users (with updated format) 2. GBrowse: Re-set up 3. JBrowse: Re-set up 4. Biomart: data re-import 5. Intermine: (internal developments) | Data refresh on portals of our data alliances and third party tools. |
| **Step 5**:   Post-release updates | 1. Update “QTL Gene” IDs from NCBI | To complete the new QTL/association data entries with "Gene IDs" assigned by NCBI GeneDB. |
